# Myosin 1g and 1f: A Prospective Analysis in NK Cell Functions

**DOI:** 10.3389/fimmu.2021.760290

**Published:** 2021-12-14

**Authors:** David Cruz-Zárate, Carlos Emilio Miguel-Rodríguez, Irving Ulises Martínez-Vargas, Leopoldo Santos-Argumedo

**Affiliations:** ^1^ Departamento de Biomedicina Molecular, Centro de Investigación y de Estudios Avanzados del Instituto Politécnico Nacional, Ciudad de México, Mexico; ^2^ Departamento de Inmunología, Escuela Nacional de Ciencias Biológicas, Instituto Politécnico Nacional, Ciudad de México, Mexico; ^3^ Departamento de Infectómica y Patogénesis Molecular, Centro de Investigación y de Estudios Avanzados del Instituto Politécnico Nacional, Ciudad de México, Mexico

**Keywords:** Class I Myosin, NK cell, Myo1g, Myo1f, cytoskeleton

## Abstract

NK cells are contained in the ILC1 group; they are recognized for their antiviral and antitumor cytotoxic capacity; NK cells also participate in other immune response processes through cytokines secretion. However, the mechanisms that regulate these functions are poorly understood since NK cells are not as abundant as other lymphocytes, which has made them difficult to study. Using public databases, we identified that NK cells express mRNA encoding class I myosins, among which Myosin 1g and Myosin 1f are prominent. Therefore, this mini-review aims to generate a model of the probable participation of Myosin 1g and 1f in NK cells, based on information reported about the function of these myosins in other leukocytes.

## Introduction

Innate lymphoid cells (ILCs) neither express T and B lymphocyte receptors but are derived from common lymphoid progenitors (CLPs) (1, 2). There is evidence about the capacity of ILC2 and ILC3 needed to recognize and present antigen to T lymphocytes and, in this way, maintain immune homeostasis (3–6). ILCs have been considered the innate equivalent of T helper lymphocytes (Th), Th1, Th2, and Th17 since ILC releases the same cytokine profile of Th cell (7–10). ILCs can mirror even T regs functions due to their capacity to produce TGF-β and IL-10 (11–13). NK cells belong to the ILC1 group (14). They are crucial in antiviral and antitumor response through their cytotoxic activity (15, 16). NK cells require the optimal function of the actin cytoskeleton and cellular membrane dynamics to perform their functions. In NK cells, the actin cytoskeleton reorganization is achieved by activation signals through several activation receptors, such as; Killer-cell Immunoglobulin-like Receptor (KIR), Natural Cytotoxicity Receptor (NCR), CD16, Signaling Lymphocyte Activation Molecule (SLAM), and others (17, 18). In a reductionist model, CD16, NKp46, NKp30 receptors associate with adapter proteins with Immunoreceptor Tyrosine-based Activation (ITAM) domains such as CD3ζ, FcεRIγ; whereas NKp44 associate with DAP12. Src kinase family members phosphorylate tyrosines in the ITAMs. Phosphorylated ITAMs form a binding site for the Src homology 2 (SH2) domains of the ZAP70 and SYK tyrosine kinase, which induce SLP-76 phosphorylation. Vav1 then recognizes phosphorylated SLP-76 *via* SH2 domain (19, 20). Next, SLAM family receptors transmit activation signals through the SLAM-Associated Protein (SAP), which recruits tyrosine kinase (Fyn) (21). Then Fyn induces Vav1 phosphorylation (22). ITAM independent signaling through the NKG2D receptor also induces Vav1 recruitment *via* PI3K and Grb2 after DAP10 tyrosine phosphorylation (23–26). In this way, Vav1 has an essential role in NK cell function. Vav1-deficient NK cells show defects in tumor cell killing (27). A synergistic effect is required to achieve ubiquitin ligase c-Cbl inhibition, which controls the availability by Vav1 (28). Consequently, Vav1 regulates actin cytoskeleton polymerization by activating the GTPases Rac, Rho, and Cdc42 since Vav1 has GEF properties (29). In this dynamic process, myosins participate at different levels, either during the polarization or aggregation of integrins, maintaining membrane tension, or interacting directly with other proteins.

NK cells are not abundant as other lymphocytes; this scarcity hinders the analysis of NK functions. Searching in databases and the analysis of the mechanisms reported in other similar cells could help understand NK lymphocytes that eventually will lead to a broader perspective about the function of these cells.

## Overview of NK Cells

Natural Killer cells are innate lymphocytes (ILC1s) known primarily for their antiviral and antitumor cytotoxic capacity (16, 30). However, they also have effector functions such as releasing cytokines, such as IFN-γ, TNF-α, IL-10, and others (8, 10, 31). Thus, NK lymphocytes are considered part of the sentinels of the innate immune system. In humans, two populations of NK cells have been described, CD56^dim^CD16^+^ and CD56^bright^ CD16^dim^ (32, 33). There are differences between both populations; for example, CD56^dim^CD16^+^ has more cytotoxic capacity than CD56^bright^ CD16^dim^ or CD16^-^.

In contrast, upon monocytes-derived-stimuli, the CD56^bright^ CD16^dim/-^ NK lymphocytes release a high amount of cytokines (7, 32, 33). Thus, CD56^-^CD16^+^ subpopulation is usually found in HIV-infected individuals presenting the high expression of NK inhibitory receptors, associated with poor cytotoxic activity (34). Regarding their anatomical distribution, the presence of NK cells has been observed in both lymphoid and non-lymphoid tissues (7). The cytotoxic activity of NK lymphocytes depends on their ability to release preformed cytotoxic granules contained in vesicles (35). The exocytosis of lytic granules begins with the contact between NK lymphocyte and target cells, which gives rise to the cytotoxic synapse (36–38).

Furthermore, NK cells express on their surface receptors of the TNF family, such as FasL and TRAIL, which can induce apoptosis by binding to their Fas or TRAIL ligand, respectively (37). Thus, the regulation of NK cell functions depends on the balance between activation and inhibition signals given by receptors present on their membrane (35). Within the group of inhibitory receptors, one can find the KIRs in humans and Ly49 Isoforms (A, B, C, E, G, Q) in mice. These receptors inhibit, inside-out and outside-in, LFA-1 signaling at different levels, preventing polarization and degranulation (39, 40). Therefore, a decrease in MHC-I expression reduces the inhibitory signal and promotes the activation of the NK cell. Additionally, in both humans and mice, the CD94/NKG2A heterodimer recognizes non-classical MHC-I molecules in the context of HLA-E (human) or H2-Qa1 (mouse). The ligands of the activating receptor NKG2D are represented by MICA/B and by ULBP in humans, and Mult1 and Rae1 in mouse (7, 10, 41).

On the other hand, activation signals are given by activation receptors, for example, Ly49 (D, H, L) and KIR isoforms, NKG2D, and natural cytotoxic receptors such as NKp30 and NKp44 in humans and NKp46 in humans and mice (7, 10). Additionally, LFA-1, β1, and β2 integrins can also regulate NK cell function (42), which we will address later. Signaling of activation and inhibition receptors regulate several NK cells functions, for example, degranulation, morphological modifications to increase NK-target cell contacts, cell migration, and cytokine release. Since Myo1g and Myo1f are involved in morphological changes and vesicular traffic, studying these proteins in the NK cell physiology becomes relevant (43). Thus, the functions of NK lymphocytes are dynamic processes that may be regulated by cytoskeletal proteins such as myosins.

Furthermore, there are functional differences among NK cell subpopulations depending on their anatomical distribution (44). For example, IL-12- and IL-18-induced IFN-γ production varies between mouse CD27^high^ spleen-resident and CD27^low^ lung-resident NK cells (32). In humans, NK CD56^bright^ under *in vitro* stimulation of IL-12 and IL-18 induce the release of more IFN-γ and TNF-β than NK CD56^dim^ cell (45). Therefore, a detailed understanding of the intrinsic factors regulating NK cell functions could provide tools to modulate any particular function depending on the type of required response.

## Class I Myosins

**Figure 1:**
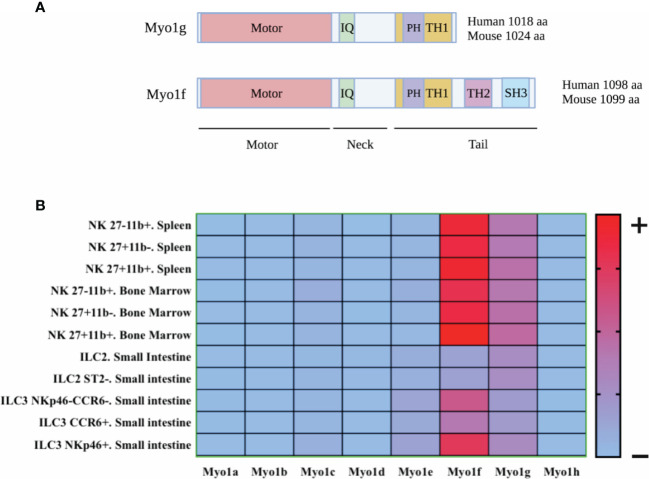
Schematic representation of Myo1g and Myo1f structure and heat map of expression of class I myosin in innate lymphoid cells. **(A)** Myo1g has a sequence of 1018 amino acids (human) and 1024 amino acids (mouse), while Myo1f has a sequence of 1098 amino acids (human) and 1099 amino acids (mouse). In addition, both myosins have a motor domain in the amino-terminal region, following a neck region with an IQ domain and a TH1 domain in the tail region which allows phosphoinositide interaction in a PH-manner dependent. Finally, Myo1f had two additional domains: TH2 (a proline-rich region) and SH3 (a proline-rich-interacting region), allowing protein-proteins interactions. Created with BioRender.com. **(B)** Class I Myosin expression in ILCs was explored with data from: http://www.immgen.org. The heat map was generated using GraphPad Prism version 8.0.0 for Mac OS X, GraphPad Software, San Diego, California USA, www.graphpad.com.

Myosins are a family of motor proteins, which are mainly known for their function in cell contractility. However, some members of this family proteins, for example, Myosin V, VI, and Ic participate in moving different cargos along the actin filaments, such as vesicles, mitochondria, and ribonuclear protein particles (46–51). Currently, 35 classes of myosins have been reported in eukaryotic organisms (52). This classification varies depending on the species; for example, 12 classes of myosin’s are described in humans (53–55). Class I myosins are non-filamentous myosins, consisting of one heavy chain and a variable number of light chains (56). The heavy chain contains three conserved regions; the ATP-dependent globular or motor domain, which binds to F-actin (57, 58). Adjacent to the motor domain is the neck region, where light chains associate and regulate the globular domain (57). In addition, the neck region has IQ domains, sequences that interact with calmodulin and calmodulin-like chains (57). Finally, the tail variable section bestows different functions depending on the domains present in that region (50, 51, 54, 56, 57). Class I myosins are subdivided into short-tailed and long-tailed myosins; both have a Pleckstrin homology domain inside the TH1 domain, as shown in **Figure 1A**, which allows interaction with several phospholipids present in the membrane and other compartments in a PH-dependent manner (51, 59). Long-tailed myosins have two additional domains; a proline-rich domain (TH2) and a domain homologous to SRC kinase (SH3) (50, 51, 56, 60). Humans and mice have a total of 8 genes coding for six short-tailed myosins (*Myo1a*, *b*, *c*, *d*, *g*, and *h*) and two long-tailed myosins (*Myo1e*, *f*) (58). Remarkably, only Myo1c, d, e, f, and g have been described in leukocytes (51, 60). Myo1g has a length of 1018 amino acids in humans and 1024 amino acids in mice (https://www.uniprot.org/) and belongs to the group of short-tailed myosins. It has a PH-type domain in the tail region, which allows its binding to lipids in the plasma membrane and microdomains rich in phospholipids and cholesterol, known as lipid rafts (59, 61, 62). The expression of Myo1g has been observed mainly in T and B lymphocytes and mast cells (63–65). Myo1g has been proposed as a bridge that allows the adequate interaction between the membrane and the cytoskeleton in processes such as cytokine secretion, cell migration, mobilization, recycling of membrane molecules, and regulating modifications in the cytoskeleton that favor cell adhesion (63, 65, 66).

On the other hand, Myo1f has a length of 1098 amino acids in humans and 1099 amino acids in mice (https://www.uniprot.org/). Its expression has been confirmed in neutrophils, macrophages, mast cells, and T lymphocytes (67–71). Similar to Myo1g, Myo1f is located adjacent to the plasma membrane, co-localizing with cortical actin and interacting with membrane phosphoinositides (67). As mentioned above, Myo1f has two additional domains, a TH2 and an SH3 that allows the interaction with several proteins. In addition, it has been observed that Myo1f interacts with 3BP2 (69), activating Cdc42 (72) suggesting a Vav1 pathway that potentially activates Rac and RhoA (73), thus regulating the cytoskeletal machinery to favor morphological changes and the generation of membrane protrusions.

As shown in **Figure 1B**, some class I myosins’ mRNAs are expressed by NK cells (74). However, it will be necessary to prove this expression at a protein level. The presence of Myosin 1g (Myo1g) and Myosin 1f (Myo1f) mRNA occur from the early stages of NK cell development (https://gexc.riken.jp). Likewise, human peripheral blood NK cells also show high Myo1g and Myo1f mRNA (75–77) (https://www.proteinatlas.org). Interestingly, tumor resident ILC1 and NK cells also express Myo1g and Myo1f (78). These results suggest that both mouse and human NK cells express both myosins and that the expression is maintained in the context of their antitumor effect. Therefore, it would be essential to analyze the role of these myosins in NK-cell differentiation, development, and functions. Class I myosins reported in leukocytes regulate processes requiring the interaction between the plasma membrane and actin cytoskeleton, such as cytokines secretion, cell migration, and mobilization of plasma membrane molecules. Therefore, we aim to analyze class I myosins’ participation in NKs functions, using information derived from results published in other leukocytes.

## Myo1g and Myo1f Could Regulate Cytotoxic Synapse Through Morphological Changes

Cytotoxicity is one of the main functions depending on morphological changes regulated by the cytoskeleton. The cytotoxic activity of NK lymphocytes first requires interaction with their target through a cytotoxic synapse and subsequently the release of cytotoxic granules towards the target cell (79). The synapse is dependent on the mobilization of different surface molecules such as adhesion molecules and integrins (80). First, the synapse requires close contact with the target cell by generating projections (filopodia and lamellipodia), this depends on the force generated by the myosins (81). The formation of these protuberances depends on Cdc42 and RhoA (29). Physical properties such as membrane tension allow membrane deformation to generate these projections (81, 82). Myo1g is abundantly expressed in the protuberances generated by B lymphocytes (63).

On the other hand, B lymphocytes show reduced membrane tension in its absence, decreasing their ability to generate filopodia and lamellipodia (83). Besides, *Myo1f* siRNA-treated macrophages decrease their capacity to generate morphological changes (84). In this way, Myo1g and Myo1f could participate in the early stages of the cytotoxic synapse of the NK cell, regulating the formation of membrane protrusions that allow interaction with their target cell.

## Probable Participation of Myo1g and Myo1f in NK Cell Migration

Cell migration depends on cytoskeleton changes that promotes the interaction of migrating cells with the endothelium. There is evidence showing that class I myosins regulate the expression of the molecules during leukocyte migration (65–67, 85). NK cells are recruited to different tissue compartments, i.e., lymph nodes and inflamed tissues, where they perform different functions such as promoting DC maturation, T cell polarization, and as cytotoxic effector cells (86, 87). NK cells express β1, β2, and β7 integrins, PSGL-1, CD62L, and various chemokine receptors such as CXCR1, CXCR2, CXCR4, CCR5 y CCR7, which allow their interaction with HEV during lymph nodes (86, 88, 89). NK subpopulations in humans and mice show differences in the expression levels of integrins and adhesion molecules (86). Therefore, the mechanisms by which NK cells migrate to different anatomical sites are not yet fully understood. Selectins and integrins regulate the interaction between the cell and the endothelium, so the expression of these molecules and their mobilization is essential during cell migration. Myo1g-deficient B lymphocytes have reduced adhesion to the endothelium due to a lower expression of LFA-1, CD62L and, VLA-4 (65).

Moreover, in the absence of Myo1g, B lymphocytes have a lower capacity for CXCL13-dependent transmigration to the inguinal node, furthermore *in vitro* CXCL12-dependent migration is also reduced (65). Migration defects were attributed to a decrease in the expression of adhesion molecules and a lower capacity to generate morphological changes due to the absence of Myo1g. Myo1f-deficient mice showed a reduction in the recruitment of neutrophils in a lung damage model (68). These neutrophils did not present defects in rolling and adhesion but in extravasation *in vivo*, explained by inefficient nucleus deformation during migration (68). *In vitro* CXCL1-dependent chemotaxis was also affected (68). Although it has not been observed that Myo1f participates directly in cell migration, it has been seen that Myo1f affects the expression of integrins β1 and β7 in mast cells (72).

Additionally, in mast cells, the activation of phosphatidylinositol 3-kinase (PI3K) increases PI(3,4,5)P3, causing the recruitment and association of 3BP2 with Myo1f during KIT activation (69). Although the consequence of the interaction of both proteins in other cell types has not been evaluated, in mast cells, 3BP2 participates in different processes; such as degranulation, by regulating the SYK, LAT, and PLC-γ pathway; in survival, by regulating the KIT, STAT1, Akt and ERK pathway; and during cell migration, by activating the Cdc42 and Rac2 pathway and regulating the expression of integrin β1 (69). Furthermore, 3BP2 is essential for activating Vav1 (73), impacting the activation of GTPases of the Rho family. The absence of Myo1f impacts the activation of Cdc42 (72), then its association with 3BP2 could play a role in the activation of Vav1. Consequently, the activation of the GTPases of the Rho family, essential in the polymerization of the actin cytoskeleton, will be affected. RhoA controls the polymerization of cortical actin through its interaction with ROCK1 and ROCK2, forming stress fibers (90). Rac1 and Rac2 are involved in the polymerization of the actin cytoskeleton *via* the SCAR/WAVE effectors, while Cdc42 controls cell polarity for migration, synapse formation, and cytokine secretion *via* effectors of the WASP family (29, 91). The role played by Myo1f, and 1g could be crucial for the migration of NK lymphocytes since they could participate by independent mechanisms due to their structural differences.

## Myo1g and Myo1f Regulate Adhesion Molecules Expression in Leukocytes

Adhesion molecules such as selectins and integrins, in addition to regulating the migration, and increasing the adhesion during cytotoxic synapse, also participate in the activation of NK cells. β1 and β2 integrins regulate the interaction of the NK cell synapsis, while LFA-1 participates in the polarization of cytotoxic granules and increasing adhesion during synapse (92, 93). As an example of its importance in other lymphocytes, Myo1g-deficient B cells have reduced CD62L and LFA-1 (65). It has been speculated that Myo1g participates in the vesicular trafficking of these molecules (66). However, no significant differences in LFA-1 expression were found in Myo1g-deficient T cells (64). Thus, it is necessary first to evaluate LFA-1 expression and other adhesion molecules in Myo1g-deficient NK cells. LFA-1 has acquired notoriety in NK cells because it participates in the activation, adhesion, and regulation of cytotoxic granules (38).

Similarly, Myo1f is crucial for the expression of integrins in leukocytes. Myo1f-deficient neutrophils increase the expression of β2-integrin, which enhances their adherence to ICAM-1 (67). Additionally, macrophage cell lines such as RAW 264.7 and J774, with stable overexpression of Myo1f-GFP, have an increased expression of integrin αVβ3, leading to increased adhesion vitronectin and promoting an inflammatory phenotype *via* ILK/Akt/mTOR activation (70). Silencing Myo1f in human mast cells negatively impacts the expression of the integrin β1 and β7, affecting exocytosis (72). Therefore, the role of Myo1f may be highly relevant for the cytotoxicity of NK cells because it is plausible to think that it may regulate the expression of integrins or other membrane molecules essential in NK cell activation.

## Myo1g and Myo1f Could Regulate NK Cell Cytotoxicity

Unlike cytotoxic T lymphocytes, NK cells have performed cytotoxic granules (31). Thus, NK cells have a faster cytotoxic activity, which becomes relevant in viral infections where a quick response is required (94, 95). Once the cell recognizes its target, these granules are mobilized to the synapse site by the mTOC (96). Then, these granules fuse with the cell membrane and are released into the pocket of the synapse (97, 98). Thus, LFA-1 primarily mediates tight maintenance of the synaptic cleft (92). In this regard, Myo1f has been reported to participate during granule mobilization in mast cells through a mechanism dependent on Cdc42 activation (72). However, it has not been evaluated whether Myo1g could have a similar function in activating GTPases of the Rho family (69, 72).

## Participation of Myo1g and Myo1f in Cytokine Production and Release

NK cells produce and secrete IFN-γ and TNF-α (7). It has been reported that IFN-γ production in infections by murine norovirus depends on ISG15 signaling (99–101). The binding of ISG15 to LFA-1 strongly induces the production of IFN-γ and IL-10 (99–101). The absence of Myo1g decreases LFA-1 expression in B lymphocytes (63, 65), suggesting that Myo1g could participate in the LFA-1-dependent IFN-γ production in the context of viral infections. Cytokines release depends on the fusion of secretory vesicles with the plasma membrane, resulting in the content release towards extracellular space (102). Diverse reports have shown the participation of Myo1g and Myo1f in releasing TNF-α, IL-6, IL-1, lactoferrin, IFN-γ, and prolactin in B lymphocytes, neutrophils, and mast cells (63, 67, 70, 72). Whether Myo1g and Myo1f are required for cytokines released by NK cells waits to be determined. However, accumulated evidence with other leukocytes points out in that direction.

## Myo1g and Myo1f Could Regulate Other Essential NK Cell Functions

Myo1g, through its PH domain, participates in mobilizing and recycling lipid rafts, indirectly moving molecules, such as CD44 (66, 103). Lipid rafts from NK cells’ membrane are mobilized to the contact site of target cells, but they are excluded in cells resistant to lysis (104). It has been suggested that signaling the KIR2DL1 protein in the cytotoxic synapse inhibits the polarization of the lipid rafts, thus preventing the death of the target cell (104). Given the role of Myo1g in mobilizing lipid rafts (66), it is likely that it participates in mobilizing these microdomains during activation and inhibition of NK-cell cytotoxicity. For this reason, it would be interesting to analyze whether Myo1g has a similar function during NK-cell lipid rafts mobilization during synapsis and in other functions, where lipid microdomains mobilization is also required. Besides, it has been reported that in the absence of Myo1g, lymphocytes present a lower membrane tension, which decreases their ability to generate membrane structures (64, 83). In addition to regulating the elasticity and stiffness of the membrane, membrane tension can generate morphological changes through the PLD2-mTORC2 signaling pathway (82, 105). Since, Myo1g and Myo1f are located adjacent to the plasma membrane, co-localizing with cortical actin (63, 67). So then, it would be interesting to know if Myo1g and Myo1f intervene in the mechano-transduction process by NK cells as described in other cell types.

## Discussion

**Figure 2:**
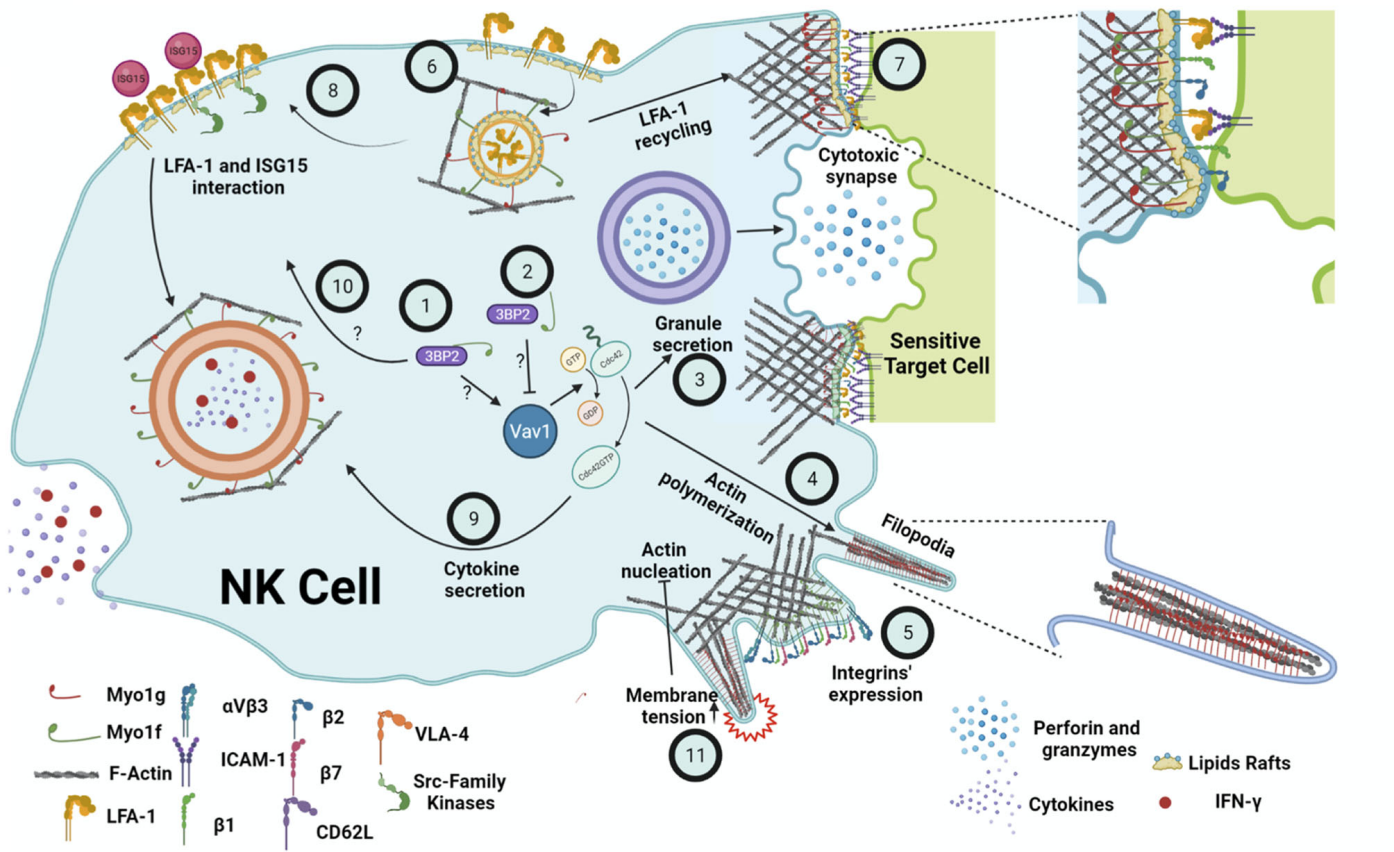
Summary of the possible functions of Myo1g and Myo1f in the NK cell. It is suggested that the interaction of Myo1f with 3BP2 causes the activation of Cdc42 through Vav1 (1). On the other hand, reduced interaction Myo1f and 3BP2 causes a decreased activation of Vav1 and, in turn, lower activation of Cdc42 (2). Consequently, less actin is polymerized during the secretion of cytotoxic granules (3), protrusions formation (4), and cytokine secretion. The participation of Myo1g and Myo1f is also essential, regulating the membrane tension. Furthermore, Myo1f deficiency causes defects in β1, β2, β7 integrins expression (5). Moreover, Myo1g-deficient B cells show lower LFA-1 membrane expression, probably due to defects in recycling (6) similar to the ones present in CD44. A decrease in LFA-1 at the cytotoxic synapse (7) will impair cytotoxic activity. Additionally, a lower amount of LFA-1 in the membrane would cause a lower interaction with ISG15 and defects in Src family kinases signaling (8), which induces the secretion of cytokines and IFN-γ, also mediated by Cdc42 (9). Therefore, it would be possible that the interaction of Myo1f and 3BP2 (10) could regulate Src; however, none of these interactions have been proven in NK cells. Finally, membrane tension negatively regulates actin polymerization through the PLD2-mTORC2 pathway (11). Thus, both Myo1g and Myo1f could regulate NK cell membrane tension and, consequently, mechano-transduction. Created with BioRender.com.

To date, there is no information about the role of class I myosins in NK cells functions. However, evidence in other cell lineages suggests that Myo1g and Myo1f could participate by regulating different NK cell functions such as cytokines release, synapse formation, granule mobilization, and migration. The functional defects described by the absence of Myo1g and Myo1f could similarly affect NK cells, causing increased susceptibility to viral infections and tumor development. Due to the tail’s structural differences, the mechanism by which Myo1g and Myo1f may regulate these processes will not be the same. Since Myo1f has a TH2 and an SH3 domain, the functions of Myo1f could depend on protein-protein interactions (85, 106), while the function of Myo1g could depend on its interaction with phosphoinositides present in membranes and vesicles (85). The function of class I myosins seems to depend on cell activation but also cell lineage. Therefore, it would be interesting to study these proteins in the context of NK cells and other ILCs subpopulations. To date, there is no information available about myosin mutations in humans that could be associated with NK cell function. However, the use of murine models or cell lines deficient or overexpressing Myo1g and Myo1f could reveal the role of these myosins in NK cells and other ILCs subpopulations. **Figure 2** summarizes what we believe may be the participation of Myo1g and Myo1f in the functions of NK cells.

## Author Contributions

DC-Z, CM-R, IM-V, and LS-A wrote the manuscript with contributions by all authors. DC-Z, CM-R, and IM-V designed at the heat map under LS-A supervision. DC-Z, CM-R, and IM-V designed and drew the illustration under LS-A supervision. All authors contributed to the article and approved the submitted version.

## Funding

DCZ, CEMR and IMV were supported by CONACyT schoolarships 632703, 780860 and 780744.

## Conflict of Interest

The authors declare that the research was conducted in the absence of any commercial or financial relationships that could be construed as a potential conflict of interest.

## Publisher’s Note

All claims expressed in this article are solely those of the authors and do not necessarily represent those of their affiliated organizations, or those of the publisher, the editors and the reviewers. Any product that may be evaluated in this article, or claim that may be made by its manufacturer, is not guaranteed or endorsed by the publisher.
